# (2*E*)-3-{4-[(1*H*-1,3-Benzimidazol-2-yl)meth­oxy]-3-eth­oxy­phen­yl}-1-(4-bromo­phen­yl)prop-2-en-1-one monohydrate

**DOI:** 10.1107/S1600536811008154

**Published:** 2011-03-12

**Authors:** Jerry P. Jasinski, William M. Miller, S. Samshuddin, B. Narayana, H. S. Yathirajan

**Affiliations:** a Department of Chemistry, Keene State College, 229 Main Street, Keene, NH 03435-2001, USA; b Department of Studies in Chemistry, Mangalore University, Mangalagangotri 574 199, India; c Department of Studies in Chemistry, University of Mysore, Manasagangotri, Mysore 570 006, India

## Abstract

In the title compound, C_25_H_21_BrN_2_O_3_·H_2_O, the benzimidazole fragment and the water mol­ecule of crystallization are each disordered over two sets of sites of equal occupancy. The dihedral angles between the least-squares planes of the benzimidazole and the 3-eth­oxy- and 4-bromo­benzene rings are 86.9 (6) and 85.1 (1)°, respectively in one disorder component. The crystal packing is stabilized by inter­molecular O—H⋯O, O—H⋯N and N—H⋯N hydrogen bonds, which link the mol­ecules into chains along the *a* axis.

## Related literature

For the biological activity of benzimidazoles, see: Pujar *et al.* (1988 ▶); Bouwman *et al.* (1990 ▶). For the use of benzimidazoles in pest control, see: Madkour *et al.* (2006 ▶). For the properties and uses of chalcones, see: Dhar (1981 ▶); Dimmock *et al.* (1999 ▶); Satyanarayana *et al.* (2004 ▶); Sarojini *et al.* (2006 ▶). For related structures, see: Jian *et al.* (2003 ▶); Odabaşoğlu *et al.* (2007 ▶). For standard bond lengths, see: Allen *et al.* (1987 ▶). 
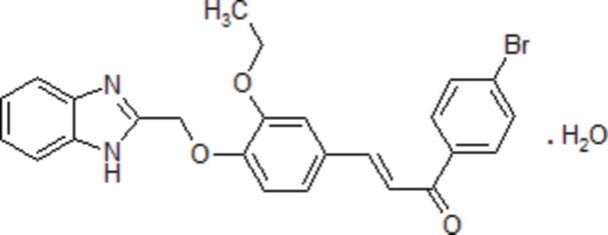


## Experimental

### 

#### Crystal data

C_25_H_21_BrN_2_O_3_·H_2_O
*M* *_r_* = 495.36
Orthorhombic, 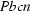

*a* = 13.8406 (12) Å
*b* = 16.5192 (8) Å
*c* = 19.4719 (13) Å
*V* = 4452.0 (5) Å^3^
*Z* = 8
Mo *K*α radiation
μ = 1.88 mm^−1^
*T* = 295 K
0.52 × 0.38 × 0.31 mm

#### Data collection

Oxford Diffraction Xcalibur Ruby Gemini diffractometer
Absorption correction: multi-scan (*CrysAlis RED*; Oxford Diffraction, 2007 ▶) *T*_min_ = 0.518, *T*_max_ = 1.000
18755 measured reflections
4033 independent reflections
2083 reflections with *I* > 2σ(*I*)
*R*_int_ = 0.073

#### Refinement

*R*[*F* ^2^ > 2σ(*F* ^2^)] = 0.058
*wR*(*F* ^2^) = 0.160
*S* = 1.04
4033 reflections
305 parameters
114 restraints
H atoms treated by a mixture of independent and constrained refinement
Δρ_max_ = 0.46 e Å^−3^
Δρ_min_ = −0.36 e Å^−3^

### 

Data collection: *CrysAlis PRO* (Oxford Diffraction, 2007 ▶); cell refinement: *CrysAlis PRO*; data reduction: *CrysAlis RED* (Oxford Diffraction, 2007 ▶); program(s) used to solve structure: *SHELXS97* (Sheldrick, 2008 ▶); program(s) used to refine structure: *SHELXL97* (Sheldrick, 2008 ▶); molecular graphics: *SHELXTL* (Sheldrick, 2008 ▶); software used to prepare material for publication: *SHELXTL*.

## Supplementary Material

Crystal structure: contains datablocks global, I. DOI: 10.1107/S1600536811008154/ez2232sup1.cif

Structure factors: contains datablocks I. DOI: 10.1107/S1600536811008154/ez2232Isup2.hkl

Additional supplementary materials:  crystallographic information; 3D view; checkCIF report

## Figures and Tables

**Table 1:** Hydrogen-bond geometry (Å, °)

*D*—H⋯*A*	*D*—H	H⋯*A*	*D*⋯*A*	*D*—H⋯*A*
N2—H2*B*⋯N2*B*^i^	0.86	2.10	2.908 (9)	157
N2*B*—H2*BA*⋯N2^i^	0.86	2.06	2.908 (9)	172
O1*WA*—H1*W*1⋯O1*WA*^ii^	0.82 (2)	1.89 (3)	2.69 (2)	164 (7)
O1*WA*—H1*W*2⋯N1	0.82 (2)	1.87 (2)	2.674 (16)	166 (6)
O1*WA*—H1*W*2⋯N1*B*	0.82 (2)	2.20 (3)	2.997 (17)	164 (6)
O1*WB*—H1*W*4⋯O1^iii^	0.82 (2)	2.33 (7)	2.912 (11)	129 (8)

Symmetry codes: (i) 
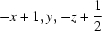
; (ii) 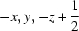; (iii) 
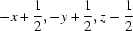
.

**Fig. 1.**
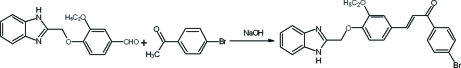
Reaction scheme for (I).

**Fig. 2.**
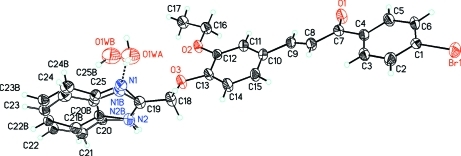
Molecular structure of the title compound showing the atom labeling scheme and 30% probability displacement ellipsoids. The disordered benzimidazol fragment has 0.50 (0) and 0.50 (0) occupancy components. Dashed lines indicate strong O—H···N intermolecular hydrogen bonds between O1WA and disordered N1 or N1B (0.50 (0) occupancy) atoms.

**Fig. 3.**
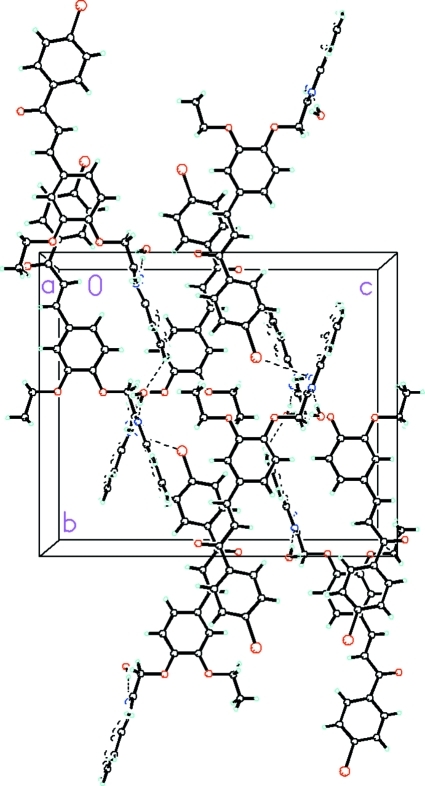
Packing diagram of the title compound viewed down the a axis. Dashed lines indicate N—H···N, O—H···O and O—H···N intermolecular hydrogen bonds linking the molecules into one-dimensional chains.

## supplementary crystallographic information

### Comment

The benzimidazole ring system and its related compounds play an important role in pharmaceutical and agricultural fields due to their broad spectrum of biological activities (Pujar *et al.*, 1988, Bouwman *et al.*, 1990). The synthesis of novel benzimidazole derivatives remains a main focus of medicinal research. Benzimidazoles are also useful as insecticides, acaricides, nematocides, herbicides and other plant-protective agents in the field of pest control (Madkour *et al.*, 2006). In recent years, attention has increasingly been given to the synthesis of benzimidazole derivatives as a source of new antimicrobial agents. In addition, benzimidazole derivatives have played a crucial role in the theoretical development of heterocyclic chemistry and are also used extensively in organic synthesis.

Chalcones constitute an important family of substances belonging to the flavonoids, a large group of natural and synthetic products with interesting physicochemical properties, biological activities and structural characteristics. Chalcones are highly reactive substances of varied nature. They have been reported to possess many interesting pharmacological activities (Dhar, 1981) including anti-inflammatory, antimicrobial, antifungal, antioxidant, cytotoxic, antitumor and anticancer activities (Dimmock *et al.*, 1999; Satyanarayana *et al.*, 2004). Chalcones are also finding application as organic nonlinear optical materials (NLO) for their SHG conversion efficiency (Sarojini *et al.*, 2006).

The crystal structures of some benzimidazole derivatives *viz*., 2-chloromethyl-1*H*-benzimidazole nitrate (Jian *et al.*, 2003) and 5-methoxy-1*H*-benzo[*d*]imidazole-2(3*H*)-thione (Odabaşoğlu *et al.*, 2007) have been reported. Encouraged by the diverse biological activities of benzimidazoles and chalcones, it was decided to prepare a new chalcone derivative of 2-aryloxy methylbenzimidazole, thus bringing both types of functional groups together in a single molecule. This paper reports the crystal structure of the title compound, C_25_H_21_N_2_O_3_Br. H_2_O, (I).

In the title compound, (I), the benzimidazole fragment and an associated water molecule are each disordered over two positions in a ratio of 0.50 (0) and 0.50 (0) (Fig. 2). The dihedral angles between the least squares planes of the benzimidazol (0.50 (0) component) and the 3-ethoxy and 4-bromo benzene rings are 86.9 (6)° and 85.1 (1)°, respectively (Fig. 3). Bond distances and angles are in normal ranges (Allen *et al.*, 1987). Crystal packing is stabilized by O—H···O, N—H···O and N—H···N intermolecular hydrogen bonds (Table 1) linking the molecules into one-dimensional chains along the *a* axis (Fig. 4).

### Experimental

A mixture of a 4-(1*H*-benzimidazol-2-ylmethoxy)-3-ethoxybenzaldehyde (0.005 mole) and *p*-bromo acetophenone (0.005 mole) in 50 ml ethanolic sodium hydroxide was stirred at 5–10°C for 3 h (Fig. 1), then maintained at room temperature for 24 h and poured into ice cold water. The precipitate that appeared after neutralization with dil. HCl was filtered off and recrystallized from 1,4-dioxane. The single crystals were grown from DMF by the slow evaporation method, with a yield of 85%. (m.p. 414 K). Analytical data: Found (Cald): C%: 62.89(62.90); H%: 4.39 (4.43); N%: 5.81 (5.87).

### Refinement

The C and N atoms in the benzimidazole fragment and O and H atoms in the water molecule are disordered and all placed at 0.50 (0) occupancy. The OW1A—H1W1, O1WA—H1W2, OW1B—H1W3, O1WB—H1W4 bond lengths were fixed at 0.82Å and the H1W1—H1W2, H1W3—H1W4 angular distances were fixed at 1.297 Å. The HN2B and H2BA atoms bonded to the disordered N2 (0.50 (0)) and N2B (0.50 (0)) atoms, respectively, were placed at their disordered sites and refined by the riding model. All of the remaining H atoms were placed in their calculated positions and then refined using the riding model with atom—H lengths of 0.93Å (CH), 0.97Å (CH_2_), 0.96Å (CH_3_) or 0.86Å (NH). Isotropic displacement parameters for these atoms were set to 1.19–1.20 (CH, CH_2_), 1.49 (CH_3_) or 1.19 (NH) times *U*_eq_ of the parent atom.

### Crystal data

**Table:**

C_25_H_21_BrN_2_O_3_·H_2_O	*F*(000) = 2032
*M**_r_* = 495.36	*D*_x_ = 1.478 Mg m^−^^3^
Orthorhombic, *P**b**c**n*	Mo *K*α radiation, λ = 0.71073 Å
Hall symbol: -P 2n 2ab	Cell parameters from 4366 reflections
*a* = 13.8406 (12) Å	θ = 5.0–32.7°
*b* = 16.5192 (8) Å	µ = 1.88 mm^−^^1^
*c* = 19.4719 (13) Å	*T* = 295 K
*V* = 4452.0 (5) Å^3^	Prism, pale yellow
*Z* = 8	0.52 × 0.38 × 0.31 mm

### Data collection

**Table:**

Oxford Diffraction Xcalibur Ruby Gemini diffractometer	4033 independent reflections
Radiation source: Enhance (Mo) X-ray Source	2083 reflections with *I* > 2σ(*I*)
graphite	*R*_int_ = 0.073
Detector resolution: 10.5081 pixels mm^-1^	θ_max_ = 25.4°, θ_min_ = 5.0°
ω scans	*h* = −16→12
Absorption correction: multi-scan (*CrysAlis RED*; Oxford Diffraction, 2007)	*k* = −19→19
*T*_min_ = 0.518, *T*_max_ = 1.000	*l* = −23→19
18755 measured reflections	

### Refinement

**Table:**

Refinement on *F*^2^	Primary atom site location: structure-invariant direct methods
Least-squares matrix: full	Secondary atom site location: difference Fourier map
*R*[*F*^2^ > 2σ(*F*^2^)] = 0.058	Hydrogen site location: inferred from neighbouring sites
*wR*(*F*^2^) = 0.160	H atoms treated by a mixture of independent and constrained refinement
*S* = 1.04	*w* = 1/[σ^2^(*F*_o_^2^) + (0.068*P*)^2^ + 2.7272*P*] where *P* = (*F*_o_^2^ + 2*F*_c_^2^)/3
4033 reflections	(Δ/σ)_max_ < 0.001
305 parameters	Δρ_max_ = 0.46 e Å^−^^3^
114 restraints	Δρ_min_ = −0.36 e Å^−^^3^

### Special details

**Table:**

Geometry. All e.s.d.'s (except the e.s.d. in the dihedral angle between two l.s. planes) are estimated using the full covariance matrix. The cell e.s.d.'s are taken into account individually in the estimation of e.s.d.'s in distances, angles and torsion angles; correlations between e.s.d.'s in cell parameters are only used when they are defined by crystal symmetry. An approximate (isotropic) treatment of cell e.s.d.'s is used for estimating e.s.d.'s involving l.s. planes.
Refinement. Refinement of *F*^2^ against ALL reflections. The weighted *R*-factor *wR* and goodness of fit *S* are based on *F*^2^, conventional *R*-factors *R* are based on *F*, with *F* set to zero for negative *F*^2^. The threshold expression of *F*^2^ > σ(*F*^2^) is used only for calculating *R*-factors(gt) *etc*. and is not relevant to the choice of reflections for refinement. *R*-factors based on *F*^2^ are statistically about twice as large as those based on *F*, and *R*- factors based on ALL data will be even larger.

### Fractional atomic coordinates and isotropic or equivalent isotropic displacement parameters (Å^2^)

**Table:**

	*x*	*y*	*z*	*U*_iso_*/*U*_eq_	Occ. (<1)
Br1	0.36419 (4)	−0.33624 (3)	0.39000 (3)	0.0699 (2)	
O1	0.3916 (3)	0.02167 (18)	0.55959 (18)	0.0678 (11)	
O2	0.3780 (2)	0.45425 (15)	0.47027 (14)	0.0491 (8)	
O3	0.3733 (3)	0.45641 (16)	0.33737 (14)	0.0564 (9)	
C1	0.3652 (3)	−0.2277 (3)	0.4205 (2)	0.0483 (12)	
C2	0.3675 (4)	−0.1650 (3)	0.3743 (3)	0.0595 (14)	
H2A	0.3669	−0.1754	0.3274	0.071*	
C3	0.3708 (4)	−0.0862 (3)	0.3983 (2)	0.0564 (14)	
H3A	0.3717	−0.0436	0.3671	0.068*	
C4	0.3727 (3)	−0.0700 (2)	0.4679 (2)	0.0440 (12)	
C5	0.3679 (4)	−0.1340 (3)	0.5130 (2)	0.0579 (14)	
H5A	0.3680	−0.1240	0.5600	0.069*	
C6	0.3630 (4)	−0.2123 (3)	0.4897 (3)	0.0569 (14)	
H6A	0.3582	−0.2548	0.5208	0.068*	
C7	0.3810 (3)	0.0138 (3)	0.4975 (3)	0.0475 (12)	
C8	0.3796 (3)	0.0841 (3)	0.4527 (3)	0.0532 (13)	
H8A	0.3674	0.0767	0.4061	0.064*	
C9	0.3949 (3)	0.1581 (2)	0.4756 (2)	0.0481 (12)	
H9A	0.4073	0.1623	0.5225	0.058*	
C10	0.3952 (3)	0.2344 (2)	0.4375 (2)	0.0443 (12)	
C11	0.3915 (3)	0.3077 (2)	0.4727 (2)	0.0453 (12)	
H11A	0.3936	0.3071	0.5204	0.054*	
C12	0.3848 (3)	0.3807 (2)	0.4397 (2)	0.0422 (12)	
C13	0.3839 (3)	0.3816 (3)	0.3674 (2)	0.0454 (12)	
C14	0.3914 (4)	0.3099 (3)	0.3323 (2)	0.0606 (15)	
H14A	0.3936	0.3107	0.2846	0.073*	
C15	0.3957 (4)	0.2361 (3)	0.3664 (2)	0.0568 (14)	
H15A	0.3989	0.1881	0.3416	0.068*	
C16	0.3816 (4)	0.4565 (3)	0.5438 (2)	0.0559 (14)	
H16A	0.4430	0.4355	0.5599	0.067*	
H16B	0.3302	0.4235	0.5630	0.067*	
C17	0.3696 (4)	0.5425 (3)	0.5656 (3)	0.0616 (15)	
H17A	0.3617	0.5449	0.6146	0.092*	
H17B	0.3137	0.5651	0.5437	0.092*	
H17C	0.4259	0.5729	0.5526	0.092*	
C18	0.3559 (4)	0.4556 (3)	0.2653 (2)	0.0596 (15)	
H18A	0.3025	0.4195	0.2550	0.072*	
H18B	0.4127	0.4361	0.2413	0.072*	
C19	0.3322 (4)	0.5392 (3)	0.2419 (2)	0.0507 (13)	
N1	0.2329 (9)	0.5577 (7)	0.2317 (6)	0.0470 (17)	0.50
N2	0.3896 (9)	0.5909 (8)	0.2252 (6)	0.0442 (16)	0.50
H2B	0.4515	0.5865	0.2258	0.053*	0.50
C20	0.3371 (4)	0.6595 (4)	0.2046 (4)	0.0440 (15)	0.50
C21	0.3602 (4)	0.7363 (4)	0.1805 (4)	0.058 (2)	0.50
H21A	0.4244	0.7528	0.1787	0.070*	0.50
C22	0.2874 (5)	0.7883 (4)	0.1589 (4)	0.048 (2)	0.50
H22A	0.3029	0.8397	0.1427	0.058*	0.50
C23	0.1915 (4)	0.7637 (4)	0.1615 (4)	0.049 (2)	0.50
H23A	0.1428	0.7985	0.1471	0.059*	0.50
C24	0.1684 (4)	0.6869 (4)	0.1857 (4)	0.055 (2)	0.50
H24A	0.1042	0.6704	0.1874	0.066*	0.50
C25	0.2412 (5)	0.6348 (4)	0.2072 (4)	0.0440 (15)	0.50
O1WA	0.0810 (8)	0.4606 (6)	0.2117 (5)	0.098 (2)	0.50
H1W1	0.036 (3)	0.468 (5)	0.239 (3)	0.148*	0.50
H1W2	0.121 (3)	0.495 (4)	0.222 (5)	0.148*	0.50
N1B	0.2526 (10)	0.5698 (8)	0.2266 (7)	0.0470 (17)	0.50
N2B	0.4086 (10)	0.5968 (8)	0.2335 (7)	0.0442 (16)	0.50
H2BA	0.4690	0.5911	0.2426	0.053*	0.50
C20B	0.3630 (5)	0.6620 (4)	0.2080 (4)	0.0440 (15)	0.50
C21B	0.3995 (4)	0.7368 (4)	0.1880 (4)	0.058 (2)	0.50
H21B	0.4656	0.7467	0.1903	0.070*	0.50
C22B	0.3372 (5)	0.7968 (4)	0.1647 (4)	0.048 (2)	0.50
H22B	0.3616	0.8469	0.1514	0.058*	0.50
C23B	0.2384 (5)	0.7820 (4)	0.1614 (4)	0.049 (2)	0.50
H23B	0.1967	0.8222	0.1458	0.059*	0.50
C24B	0.2020 (4)	0.7072 (5)	0.1813 (5)	0.055 (2)	0.50
H24B	0.1359	0.6973	0.1791	0.066*	0.50
C25B	0.2643 (5)	0.6472 (4)	0.2046 (4)	0.0440 (15)	0.50
O1WB	0.0642 (8)	0.5103 (6)	0.2033 (5)	0.098 (2)	0.50
H1W3	0.082 (6)	0.464 (2)	0.199 (4)	0.148*	0.50
H1W4	0.050 (8)	0.525 (5)	0.165 (2)	0.148*	0.50

### Atomic displacement parameters (Å^2^)

**Table:**

	*U*^11^	*U*^22^	*U*^33^	*U*^12^	*U*^13^	*U*^23^
Br1	0.0949 (4)	0.0427 (3)	0.0722 (4)	0.0038 (3)	0.0105 (3)	−0.0083 (3)
O1	0.104 (3)	0.0461 (19)	0.053 (2)	−0.0001 (19)	−0.008 (2)	0.0056 (16)
O2	0.081 (2)	0.0316 (15)	0.0344 (17)	0.0001 (15)	−0.0072 (17)	−0.0021 (13)
O3	0.102 (3)	0.0329 (15)	0.0340 (17)	−0.0041 (17)	−0.0102 (18)	0.0018 (13)
C1	0.060 (3)	0.036 (2)	0.049 (3)	0.000 (2)	0.017 (3)	0.001 (2)
C2	0.075 (4)	0.049 (3)	0.054 (3)	0.006 (3)	0.001 (3)	−0.002 (2)
C3	0.073 (4)	0.043 (2)	0.053 (3)	0.006 (3)	0.005 (3)	0.010 (2)
C4	0.046 (3)	0.039 (2)	0.048 (3)	0.001 (2)	0.000 (2)	0.004 (2)
C5	0.081 (4)	0.045 (3)	0.048 (3)	−0.001 (3)	0.005 (3)	0.006 (2)
C6	0.075 (4)	0.036 (2)	0.060 (3)	0.005 (3)	0.007 (3)	0.012 (2)
C7	0.049 (3)	0.044 (3)	0.049 (3)	0.003 (2)	0.001 (2)	0.005 (2)
C8	0.067 (4)	0.040 (2)	0.053 (3)	0.000 (2)	−0.001 (3)	0.006 (2)
C9	0.058 (3)	0.040 (2)	0.047 (3)	0.005 (2)	0.000 (2)	0.003 (2)
C10	0.049 (3)	0.035 (2)	0.048 (3)	0.004 (2)	−0.003 (2)	0.002 (2)
C11	0.059 (3)	0.039 (2)	0.038 (2)	0.003 (2)	−0.007 (2)	−0.001 (2)
C12	0.052 (3)	0.032 (2)	0.042 (3)	−0.001 (2)	0.000 (2)	−0.003 (2)
C13	0.064 (3)	0.036 (2)	0.037 (2)	−0.004 (2)	−0.005 (2)	0.006 (2)
C14	0.101 (4)	0.040 (2)	0.041 (3)	0.003 (3)	0.002 (3)	0.002 (2)
C15	0.088 (4)	0.030 (2)	0.052 (3)	0.004 (2)	0.003 (3)	−0.003 (2)
C16	0.075 (4)	0.048 (3)	0.044 (3)	−0.001 (3)	−0.001 (3)	−0.002 (2)
C17	0.086 (4)	0.050 (3)	0.049 (3)	−0.004 (3)	−0.004 (3)	−0.008 (2)
C18	0.092 (4)	0.041 (2)	0.046 (3)	−0.002 (3)	−0.012 (3)	0.005 (2)
C19	0.069 (4)	0.042 (3)	0.041 (3)	−0.002 (3)	−0.006 (3)	−0.005 (2)
N1	0.051 (4)	0.049 (3)	0.042 (2)	−0.004 (3)	−0.003 (3)	0.000 (2)
N2	0.046 (4)	0.038 (2)	0.049 (3)	0.001 (2)	−0.007 (3)	0.000 (2)
C20	0.060 (4)	0.040 (2)	0.031 (2)	0.005 (3)	0.001 (3)	0.000 (2)
C21	0.064 (5)	0.055 (3)	0.055 (3)	−0.002 (4)	0.009 (4)	0.000 (3)
C22	0.061 (6)	0.040 (3)	0.045 (3)	0.008 (4)	0.005 (4)	0.001 (3)
C23	0.064 (5)	0.048 (4)	0.035 (3)	0.003 (4)	0.002 (4)	−0.001 (3)
C24	0.059 (4)	0.070 (4)	0.036 (3)	0.003 (4)	−0.005 (3)	−0.014 (3)
C25	0.053 (4)	0.053 (3)	0.026 (2)	0.013 (3)	−0.007 (3)	−0.008 (2)
O1WA	0.090 (4)	0.111 (5)	0.094 (4)	−0.011 (4)	0.004 (3)	−0.003 (4)
N1B	0.051 (4)	0.049 (3)	0.042 (2)	−0.004 (3)	−0.003 (3)	0.000 (2)
N2B	0.046 (4)	0.038 (2)	0.049 (3)	0.001 (2)	−0.007 (3)	0.000 (2)
C20B	0.060 (4)	0.040 (2)	0.031 (2)	0.005 (3)	0.001 (3)	0.000 (2)
C21B	0.064 (5)	0.055 (3)	0.055 (3)	−0.002 (4)	0.009 (4)	0.000 (3)
C22B	0.061 (6)	0.040 (3)	0.045 (3)	0.008 (4)	0.005 (4)	0.001 (3)
C23B	0.064 (5)	0.048 (4)	0.035 (3)	0.003 (4)	0.002 (4)	−0.001 (3)
C24B	0.059 (4)	0.070 (4)	0.036 (3)	0.003 (4)	−0.005 (3)	−0.014 (3)
C25B	0.053 (4)	0.053 (3)	0.026 (2)	0.013 (3)	−0.007 (3)	−0.008 (2)
O1WB	0.090 (4)	0.111 (5)	0.094 (4)	−0.011 (4)	0.004 (3)	−0.003 (4)

### Geometric parameters (Å, °)

**Table:**

Br1—C1	1.888 (4)	C18—H18B	0.9700
O1—C7	1.225 (5)	C19—N2	1.212 (13)
O2—C12	1.356 (5)	C19—N1B	1.249 (14)
O2—C16	1.433 (5)	C19—N1	1.422 (14)
O3—C13	1.375 (5)	C19—N2B	1.431 (14)
O3—C18	1.424 (5)	N1—C25	1.365 (13)
C1—C2	1.372 (6)	N2—C20	1.404 (14)
C1—C6	1.372 (6)	N2—H2B	0.8600
C2—C3	1.383 (6)	C20—C21	1.3900
C2—H2A	0.9300	C20—C25	1.3900
C3—C4	1.382 (6)	C21—C22	1.3900
C3—H3A	0.9300	C21—H21A	0.9300
C4—C5	1.375 (6)	C22—C23	1.3900
C4—C7	1.504 (6)	C22—H22A	0.9300
C5—C6	1.373 (6)	C23—C24	1.3900
C5—H5A	0.9300	C23—H23A	0.9300
C6—H6A	0.9300	C24—C25	1.3900
C7—C8	1.452 (6)	C24—H24A	0.9300
C8—C9	1.318 (6)	O1WA—H1W1	0.82 (2)
C8—H8A	0.9300	O1WA—H1W2	0.82 (2)
C9—C10	1.463 (6)	O1WA—H1W3	0.25 (9)
C9—H9A	0.9300	O1WA—H1W4	1.47 (4)
C10—C15	1.385 (7)	N1B—C25B	1.359 (14)
C10—C11	1.391 (6)	N2B—C20B	1.343 (15)
C11—C12	1.370 (6)	N2B—H2BA	0.8600
C11—H11A	0.9300	C20B—C21B	1.3900
C12—C13	1.407 (6)	C20B—C25B	1.3900
C13—C14	1.371 (6)	C21B—C22B	1.3900
C14—C15	1.390 (6)	C21B—H21B	0.9300
C14—H14A	0.9300	C22B—C23B	1.3900
C15—H15A	0.9300	C22B—H22B	0.9300
C16—C17	1.493 (6)	C23B—C24B	1.3900
C16—H16A	0.9700	C23B—H23B	0.9300
C16—H16B	0.9700	C24B—C25B	1.3900
C17—H17A	0.9600	C24B—H24B	0.9300
C17—H17B	0.9600	O1WB—H1W1	1.05 (7)
C17—H17C	0.9600	O1WB—H1W2	0.90 (8)
C18—C19	1.490 (6)	O1WB—H1W3	0.82 (2)
C18—H18A	0.9700	O1WB—H1W4	0.82 (2)
			
C12—O2—C16	117.4 (3)	N2—C19—N1	116.4 (8)
C13—O3—C18	115.4 (3)	N1B—C19—N1	13.2 (10)
C2—C1—C6	120.3 (4)	N2—C19—N2B	10.3 (11)
C2—C1—Br1	120.7 (4)	N1B—C19—N2B	110.8 (9)
C6—C1—Br1	119.0 (3)	N1—C19—N2B	123.7 (8)
C1—C2—C3	119.4 (5)	N2—C19—C18	126.3 (8)
C1—C2—H2A	120.3	N1B—C19—C18	129.9 (7)
C3—C2—H2A	120.3	N1—C19—C18	117.0 (6)
C4—C3—C2	120.9 (4)	N2B—C19—C18	119.3 (7)
C4—C3—H3A	119.5	C25—N1—C19	99.7 (8)
C2—C3—H3A	119.5	C19—N2—C20	107.9 (10)
C5—C4—C3	118.4 (4)	C19—N2—H2B	126.1
C5—C4—C7	117.8 (4)	C20—N2—H2B	126.1
C3—C4—C7	123.8 (4)	C21—C20—C25	120.0
C6—C5—C4	121.0 (4)	C21—C20—N2	135.5 (7)
C6—C5—H5A	119.5	C25—C20—N2	104.3 (6)
C4—C5—H5A	119.5	C22—C21—C20	120.0
C1—C6—C5	119.9 (4)	C22—C21—H21A	120.0
C1—C6—H6A	120.0	C20—C21—H21A	120.0
C5—C6—H6A	120.0	C23—C22—C21	120.0
O1—C7—C8	120.7 (4)	C23—C22—H22A	120.0
O1—C7—C4	119.0 (4)	C21—C22—H22A	120.0
C8—C7—C4	120.3 (4)	C22—C23—C24	120.0
C9—C8—C7	122.3 (5)	C22—C23—H23A	120.0
C9—C8—H8A	118.8	C24—C23—H23A	120.0
C7—C8—H8A	118.8	C25—C24—C23	120.0
C8—C9—C10	128.8 (4)	C25—C24—H24A	120.0
C8—C9—H9A	115.6	C23—C24—H24A	120.0
C10—C9—H9A	115.6	N1—C25—C24	128.5 (7)
C15—C10—C11	118.3 (4)	N1—C25—C20	111.5 (7)
C15—C10—C9	121.6 (4)	C24—C25—C20	120.0
C11—C10—C9	120.0 (4)	H1W1—O1WA—H1W2	105 (3)
C12—C11—C10	122.6 (4)	H1W1—O1WA—H1W3	131 (10)
C12—C11—H11A	118.7	H1W2—O1WA—H1W3	92 (10)
C10—C11—H11A	118.7	H1W1—O1WA—H1W4	94 (7)
O2—C12—C11	126.0 (4)	H1W2—O1WA—H1W4	81 (9)
O2—C12—C13	115.4 (4)	H1W3—O1WA—H1W4	43 (8)
C11—C12—C13	118.6 (4)	C19—N1B—C25B	110.6 (10)
C14—C13—O3	124.9 (4)	C20B—N2B—C19	103.3 (10)
C14—C13—C12	119.3 (4)	C20B—N2B—H2BA	128.4
O3—C13—C12	115.8 (4)	C19—N2B—H2BA	128.4
C13—C14—C15	121.5 (4)	N2B—C20B—C21B	130.2 (7)
C13—C14—H14A	119.2	N2B—C20B—C25B	109.7 (7)
C15—C14—H14A	119.2	C21B—C20B—C25B	120.0
C10—C15—C14	119.6 (4)	C22B—C21B—C20B	120.0
C10—C15—H15A	120.2	C22B—C21B—H21B	120.0
C14—C15—H15A	120.2	C20B—C21B—H21B	120.0
O2—C16—C17	107.8 (4)	C21B—C22B—C23B	120.0
O2—C16—H16A	110.2	C21B—C22B—H22B	120.0
C17—C16—H16A	110.2	C23B—C22B—H22B	120.0
O2—C16—H16B	110.2	C24B—C23B—C22B	120.0
C17—C16—H16B	110.2	C24B—C23B—H23B	120.0
H16A—C16—H16B	108.5	C22B—C23B—H23B	120.0
C16—C17—H17A	109.5	C23B—C24B—C25B	120.0
C16—C17—H17B	109.5	C23B—C24B—H24B	120.0
H17A—C17—H17B	109.5	C25B—C24B—H24B	120.0
C16—C17—H17C	109.5	N1B—C25B—C24B	134.5 (8)
H17A—C17—H17C	109.5	N1B—C25B—C20B	105.5 (8)
H17B—C17—H17C	109.5	C24B—C25B—C20B	120.0
O3—C18—C19	109.2 (4)	H1W1—O1WB—H1W2	83 (6)
O3—C18—H18A	109.8	H1W1—O1WB—H1W3	64 (7)
C19—C18—H18A	109.8	H1W2—O1WB—H1W3	60 (7)
O3—C18—H18B	109.8	H1W1—O1WB—H1W4	136 (9)
C19—C18—H18B	109.8	H1W2—O1WB—H1W4	131 (10)
H18A—C18—H18B	108.3	H1W3—O1WB—H1W4	106 (3)
N2—C19—N1B	103.2 (9)		
			
C6—C1—C2—C3	2.1 (7)	N2B—C19—N1—C25	6.0 (13)
Br1—C1—C2—C3	−178.3 (4)	C18—C19—N1—C25	−176.4 (6)
C1—C2—C3—C4	0.6 (8)	N1B—C19—N2—C20	6.0 (12)
C2—C3—C4—C5	−2.2 (7)	N1—C19—N2—C20	4.7 (13)
C2—C3—C4—C7	177.0 (4)	N2B—C19—N2—C20	−132 (7)
C3—C4—C5—C6	1.1 (7)	C18—C19—N2—C20	178.0 (6)
C7—C4—C5—C6	−178.1 (4)	C19—N2—C20—C21	−179.9 (6)
C2—C1—C6—C5	−3.2 (8)	C19—N2—C20—C25	−4.9 (10)
Br1—C1—C6—C5	177.2 (4)	C25—C20—C21—C22	0.0
C4—C5—C6—C1	1.6 (8)	N2—C20—C21—C22	174.4 (10)
C5—C4—C7—O1	6.2 (7)	C20—C21—C22—C23	0.0
C3—C4—C7—O1	−172.9 (5)	C21—C22—C23—C24	0.0
C5—C4—C7—C8	−175.7 (4)	C22—C23—C24—C25	0.0
C3—C4—C7—C8	5.1 (7)	C19—N1—C25—C24	178.4 (5)
O1—C7—C8—C9	3.9 (7)	C19—N1—C25—C20	−1.0 (9)
C4—C7—C8—C9	−174.1 (4)	C23—C24—C25—N1	−179.4 (10)
C7—C8—C9—C10	−179.5 (4)	C23—C24—C25—C20	0.0
C8—C9—C10—C15	−12.1 (8)	C21—C20—C25—N1	179.5 (8)
C8—C9—C10—C11	165.7 (5)	N2—C20—C25—N1	3.5 (9)
C15—C10—C11—C12	2.4 (7)	C21—C20—C25—C24	0.0
C9—C10—C11—C12	−175.4 (4)	N2—C20—C25—C24	−176.0 (7)
C16—O2—C12—C11	2.4 (6)	N2—C19—N1B—C25B	−5.4 (13)
C16—O2—C12—C13	−178.1 (4)	N1—C19—N1B—C25B	170 (6)
C10—C11—C12—O2	177.9 (4)	N2B—C19—N1B—C25B	2.0 (13)
C10—C11—C12—C13	−1.5 (7)	C18—C19—N1B—C25B	−177.0 (6)
C18—O3—C13—C14	8.9 (7)	N2—C19—N2B—C20B	41 (6)
C18—O3—C13—C12	−169.6 (4)	N1B—C19—N2B—C20B	−3.3 (12)
O2—C12—C13—C14	179.4 (4)	N1—C19—N2B—C20B	−6.6 (13)
C11—C12—C13—C14	−1.1 (7)	C18—C19—N2B—C20B	175.8 (6)
O2—C12—C13—O3	−1.9 (6)	C19—N2B—C20B—C21B	−178.7 (5)
C11—C12—C13—O3	177.6 (4)	C19—N2B—C20B—C25B	3.2 (10)
O3—C13—C14—C15	−175.8 (5)	N2B—C20B—C21B—C22B	−177.9 (10)
C12—C13—C14—C15	2.7 (8)	C25B—C20B—C21B—C22B	0.0
C11—C10—C15—C14	−0.8 (7)	C20B—C21B—C22B—C23B	0.0
C9—C10—C15—C14	177.0 (5)	C21B—C22B—C23B—C24B	0.0
C13—C14—C15—C10	−1.8 (8)	C22B—C23B—C24B—C25B	0.0
C12—O2—C16—C17	−178.2 (4)	C19—N1B—C25B—C24B	179.4 (6)
C13—O3—C18—C19	172.1 (4)	C19—N1B—C25B—C20B	0.1 (12)
O3—C18—C19—N2	86.4 (9)	C23B—C24B—C25B—N1B	−179.2 (11)
O3—C18—C19—N1B	−103.7 (10)	C23B—C24B—C25B—C20B	0.0
O3—C18—C19—N1	−100.3 (7)	N2B—C20B—C25B—N1B	−2.3 (10)
O3—C18—C19—N2B	77.4 (8)	C21B—C20B—C25B—N1B	179.4 (8)
N2—C19—N1—C25	−2.4 (12)	N2B—C20B—C25B—C24B	178.3 (8)
N1B—C19—N1—C25	−8(4)	C21B—C20B—C25B—C24B	0.0

### Hydrogen-bond geometry (Å, °)

**Table:**

*D*—H···*A*	*D*—H	H···*A*	*D*···*A*	*D*—H···*A*
N2—H2B···N2B^i^	0.86	2.10	2.908 (9)	157
N2B—H2BA···N2^i^	0.86	2.06	2.908 (9)	172
O1WA—H1W1···O1WA^ii^	0.82 (2)	1.89 (3)	2.69 (2)	164 (7)
O1WA—H1W2···N1	0.82 (2)	1.87 (2)	2.674 (16)	166 (6)
O1WA—H1W2···N1B	0.82 (2)	2.20 (3)	2.997 (17)	164 (6)
O1WB—H1W4···O1^iii^	0.82 (2)	2.33 (7)	2.912 (11)	129 (8)

Symmetry codes: (i) −*x*+1, *y*, −*z*+1/2; (ii) −*x*, *y*, −*z*+1/2; (iii) −*x*+1/2, −*y*+1/2, *z*−1/2.
